# Arterio-biliary fistula caused by a hepatic artery pseudoaneurysm in a recently performed liver transplant: successful resolution and long-term liver implant preservation using a covered coronary stent

**DOI:** 10.1186/s42155-020-00191-6

**Published:** 2020-12-08

**Authors:** Alfredo Páez-Carpio, Elena Serrano, Federico Zarco, Constantino Fondevila, Marta Burrel

**Affiliations:** 1 Department of Radiology, CDI, Hospital Clínic, Barcelona, Spain; 2 General and Digestive Surgery Service, ICMDiM, Hospital Clínic, Barcelona, Spain

**Keywords:** Liver transplantation, Hepatic artery pseudoaneurysm, Arterio-biliary fistula, Hemobilia, Endovascular treatment, Coronary covered stent

## Abstract

**Background:**

The formation of a hepatic artery pseudoaneurysm in a liver implant is a rare but potentially fatal complication. Fistulization of such pseudoaneurysms into the bile duct is sporadic. The most common causes of hepatic artery pseudoaneurysm are infection at the anastomosis site, inadequate surgical technique, and an iatrogenic origin due to minimally invasive procedures. Currently, there is no standardized treatment in neither of these complications, with surgery and various endovascular procedures among the alternatives available. None of these therapeutic approaches has demonstrated a significant increase in long-term liver implant preservation.

**Case presentation:**

A 56-year-old man with a two-month liver transplant presented with massive upper gastrointestinal bleeding and hemobilia shortly after the performance of an endoscopic retrograde cholangiopancreatography due to the presence of a hepatic artery pseudoaneurysm with fistulization into the bile duct. This case report describes the successful treatment of both complications, the hepatic artery pseudoaneurysm and the arterio-biliary fistula, using a covered coronary stent placed in the hepatic artery. A year and a half after treatment, the patient maintains a preserved liver implant and a patent hepatic artery.

**Conclusions:**

Treatment of a hepatic artery pseudoaneurysm with fistulization into bile duct using a covered coronary stent allowed the correct repair of the defect, adequate hemorrhage control, and long-term liver implant preservation.

## Background

Hepatic artery pseudoaneurysms (HAP) are a low-incidence (0.3–2.5%) but life-threatening complication after liver transplantation, with reported mortality rates between 33% and 78% if left untreated (Marshall et al. 2001; Jain et al. 2006). Fistulization of such HAP into the bile duct is extremely rare (Stefańczyk et al. 2018). Conventional causes of HAP are infection at the anastomosis site or a poor surgical technique. However, an iatrogenic origin due to different minimally invasive procedures has emerged as a cause of HAP after liver transplantation (Marshall et al. 2001). Immediate and effective treatment is necessary in such cases, given the rapid evolution towards hemodynamic instability and the high risk of graft loss. No standard treatment exists for either complication, with no demonstrated superiority between surgical treatment and the several endovascular procedures available (Saad et al. 2013). We present a patient who recently underwent a liver transplant and presented a HAP with an arterio-biliary fistula (ABF) two months after transplantation, conditioning hemobilia, and a subsequent massive upper GI bleeding. Both complications were successfully treated placing a covered coronary stent in the liver graft’s hepatic artery, achieving long-term liver artery patency, and preserving the liver graft.

## Case report

A 56-year-old former intravenous drug user with chronic liver disease secondary to coexisting infection by hepatitis B and hepatitis C viruses was diagnosed with bifocal hepatocellular carcinoma in February 2018. After receiving bridging treatment using image-guided percutaneous liver microwave ablation and transarterial chemoembolization for each nodule, the patient received definitive treatment with an orthotopic liver transplant on January 1, 2019. The surgery went without complications, except for a non-significant size discrepancy between the native bile duct and the liver graft bile duct.

**Fig. 1:**
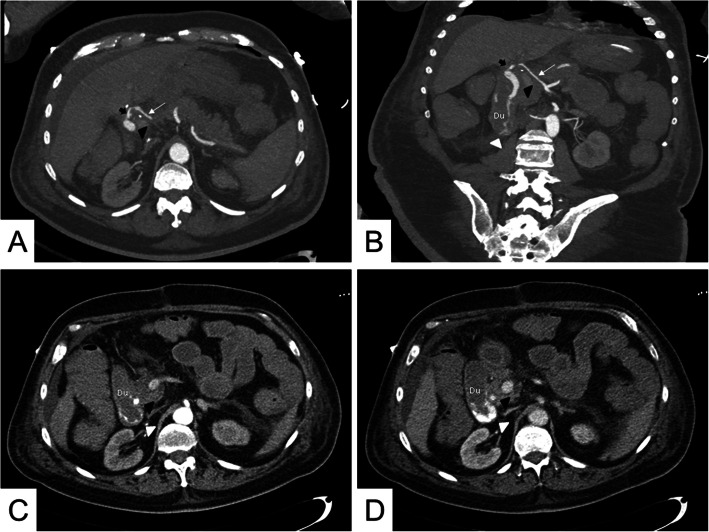
Contrast-enhanced CT showing a HAP with an ABF, hemobilia, and upper GI bleeding. **a-b** Axial and coronal sections processed with a maximum intensity projection (MIP) of a contrast-enhanced CT, showing a pseudoaneurysm (thick black arrow) from the liver graft hepatic artery (thin white arrow). Immediately adjacent is the bile duct filled with iodinated contrast (black triangle), indicating the presence of an ABF with hemobilia. **c-d** Axial section of a contrast-enhanced CT showing contrast extravasation into the duodenum (Du), which increases in quantity between the arterial phase (**c**) and the venous phase (**d**), consistent with an upper GI bleeding (white head arrow)

Nevertheless, the patient developed a stenosis of the bile duct anastomosis, which was treated 45 days later by dilating the bile duct using an expandable balloon guided by an endoscopic retrograde cholangiopancreatography (ERCP). After being discharged, the patient experienced intermittent pain in the epigastric area, which, added to a moderate amount of hematemesis and melena, led him to come to the emergency room three days later. A new ERCP was readily performed, observing significant bleeding from the duodenal papilla, unsuccessfully treated using adrenaline and argon sclerosis. Given the patient’s progressive deterioration, having to use vasoactive drugs and blood products transfusion, the attending physicians decided to refer him to our service to characterize the bleeding. A contrast-enhanced computed tomography (CT) scan was then performed, which showed a significant extravasation of contrast from the extrahepatic bile duct into the duodenum, with a smooth-walled sac adjacent and communicated to the hepatic artery in contact with the bile duct. These findings were consistent with an ABF due to a HAP (Fig. 1). Ultimately, we decided to treat the patient using endovascular therapy.

**Fig. 2:**
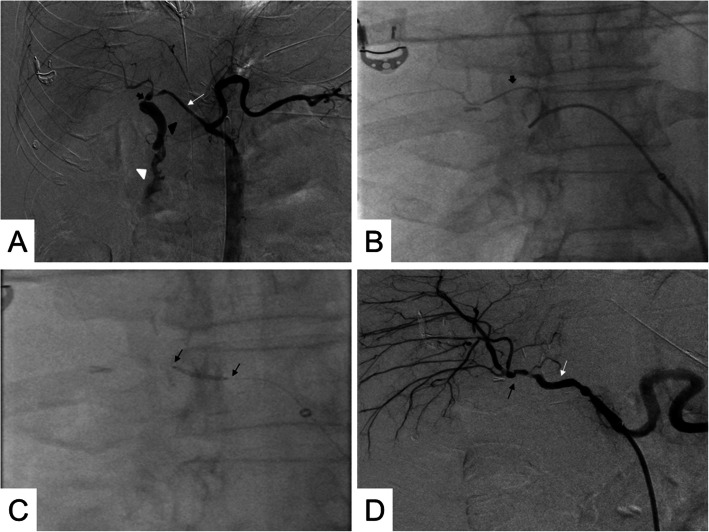
Endovascular treatment of the HAP and the ABF. **a** DSA of the celiac trunk demonstrating a HAP (thick black arrow) coming off the main hepatic artery just before its bifurcation (thin white arrow). The bile duct is filled with iodinated contrast, confirming the presence of an ABF with hemobilia (blackhead arrow). Contrast flush within the duodenum demonstrated the presence of an upper GI hemorrhage (white head arrow). **b-c** Fluoroscopic images during the positioning of the microcatheter after crossing the target lesion (thick black arrow) (**b**) and the introduction and release of a covered stent with an expandable balloon (between thin black arrows) (**c**). **d** DSA demonstrating complete exclusion of the HAP, resolution of the ABF, the permeability of the main hepatic artery (thin white arrow), and its intrahepatic branches. We noted focal stenosis in the hepatic artery and its branches, related to vasospasm and vessel remodeling after manipulation (thin black arrows)

Once the patient was under general anesthesia, and after local anesthesia administration, we accessed the right femoral artery with a 6Fr introducer (Flexor Check-Flow Introducer Set®, Cook Medical). Through the introducer, we passed a 5Fr angiographic catheter (Radifocus Angiographic Catheter®, Terumo) for a selective angiography of the celiac trunk, confirming the permeability of the liver graft’s hepatic artery and the presence of a HAP with an ABF (Fig. 2a). We then placed a 5Fr introducer sheath (Radifocus Introducer II Standard Kit®, Terumo) into the common hepatic artery and a 2.7Fr microcatheter with a 0.021” hydrophilic guide to pass through the target lesion. Then we replaced the latter with a 0.014” support guide (Hi-Torque Spartacore 14®, Abbott), placing the tip in an upper segmental branch of the right hepatic artery (Fig. 2b). Finally, we decided to place a 4.5 × 15 mm balloon-expandable covered coronary stent (PK Papyrus Covered Coronary Stent System®, Biotronik, Inc.) using a rapid exchange platform (mono-rail) with a minimum guiding catheter diameter between 5-6Fr, successfully covering both the anastomosis site and the target lesion (Fig. 2c). The last angiographic series demonstrated the correct resolution of the HAP and the ABF, as well as the patency of the hepatic artery and its intrahepatic branches (Fig. 2d).

**Fig. 3:**
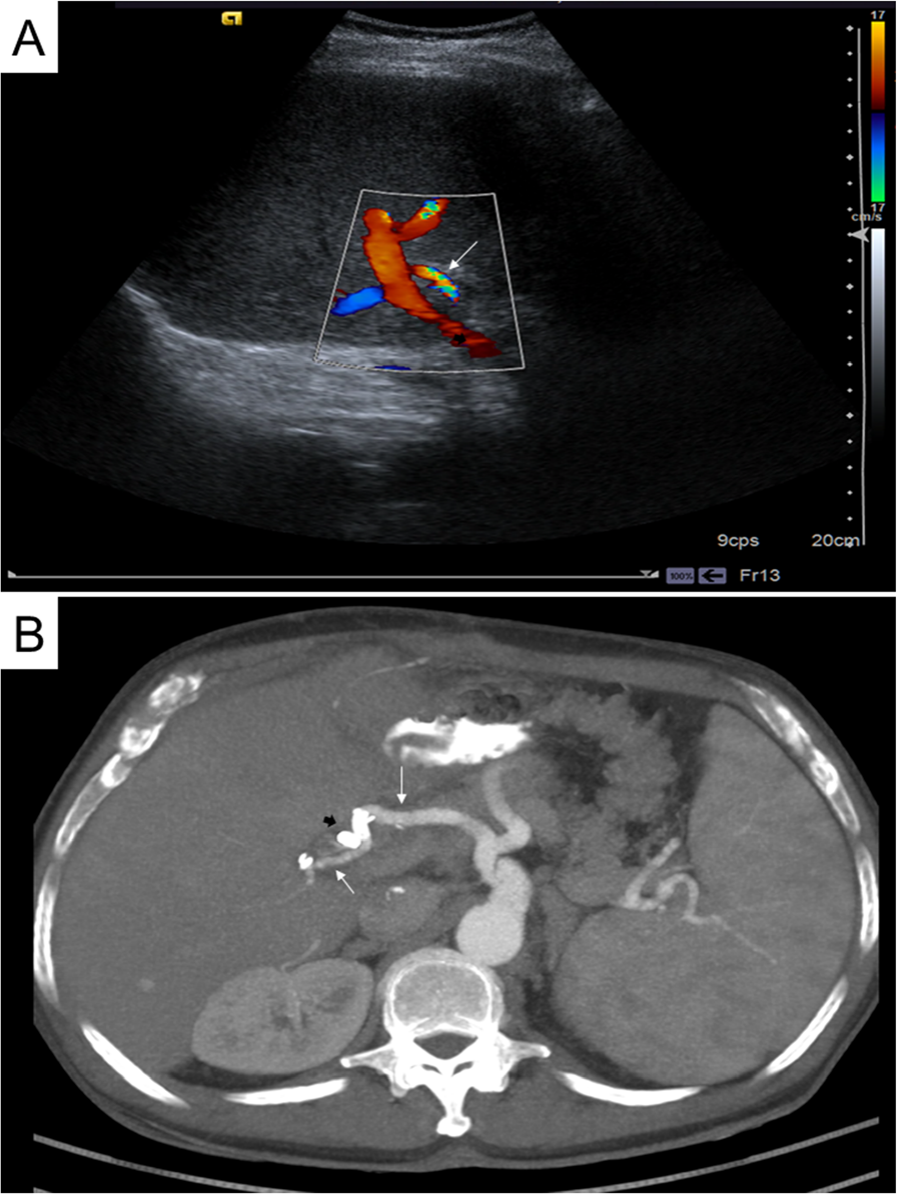
Contrast-enhanced CT and doppler ultrasound post-treatment follow-up. **a** Doppler ultrasound performed just before the patient’s discharge demonstrates the stent’s correct permeability within the hepatic artery (thin white arrow). **b** Axial section processed with a MIP of a contrast-enhanced CT in arterial phase performed 18 months after the procedure, showing the correct placement of the covered stent (thick black arrow) and hepatic artery patency (thin white arrows)

The patient presented a correct evolution during the admission, being discharged two weeks after endovascular treatment. We decided to prescribe the patient with Aspirin 100 mg/day as an antiplatelet regime during admission and indefinitely after discharge. The gastroenterology service correctly treated the biliary anastomosis stenosis by placing a biliary stent via ERCP ten weeks later, with no further complications reported. Subsequent clinical, analytical and radiological examinations demonstrated the absence of sequelae in the liver implant. Further examinations by ultrasound and CT scan up to 18 months after the procedure showed hepatic artery patency (Fig. 3).

## Discussion

Disruption of the hepatic artery in liver transplant recipients, whether termed as pseudoaneurysms or rupture, is a rare postoperative complication of liver transplantation with high morbidity and mortality. Three main etiologies are related to the appearance of HAP after liver transplantation: an inadequate surgical technique when performing the anastomosis, local infection at the anastomosis site, and as an iatrogenic complication after a minimally invasive procedure (Jain et al. 2006). Although our patient presented a HAP about 60 days after liver transplantation, coinciding with the peak incidence of the first two causes above mentioned, the presence of an ABF and a recently performed ERCP suggests an iatrogenic origin. Although the overall incidence of bleeding after ERCP is about 0.1–2%, an ABF in association with a HAP is exceptionally uncommon, and the appearance of both complications after an ERCP is anecdotal (Rustagi et al., 2015).

Surgery has conventionally been the treatment of liver artery complications after liver transplantation. However, this approach is associated with high rates of morbidity, mortality, and liver graft loss. Hence the increasing use of endovascular treatment as a therapeutic alternative (Marshall et al. 2001). Several case reports have described the treatment of these pseudoaneurysms with endovascular therapy. Among the techniques used are direct embolization of the pseudoaneurysm (Ou et al. 2011) and the use of a covered coronary stent, as in our case (Muraoka et al. 2005). Furthermore, endovascular techniques such as transcatheter embolization, direct percutaneous injection of coils or thrombin, and placement of covered stents have recently demonstrated success in treating HAP after liver transplantation in some case series (Saad et al. 2013; Pedersoli et al. 2016). Regarding the treatment of arterio-biliary fistulas, only a few case reports refer to the treatment of this complication by direct embolization of the pseudoaneurysm (Beningfield et al. 1991; Hayano et al. 2010).

In the most representative study to date, Saad et al. report on the efficacy of most endovascular techniques for HAP treatment after liver transplantation. However, they do not report significant differences in liver implant survival compared to surgical repair. Moreover, the authors indicate that one must be cautious using stent-grafts in these patients, as they require a stiffer platform that does not easily navigate through the tortuous vessels, which is quite common in liver grafts hepatic arteries. Furthermore, they report that the hepatic artery’s long-term patency in patients treated with stents may be uncertain or even reduced. (Saad et al. 2013). In our case, we decided to use a covered coronary stent due to its smaller size and more malleable platform. The latter provides better maneuverability in navigating the vascular tortuosity, allowing a faster and more comfortable placement of the stent in the hepatic artery. Treatment using a covered coronary stent solved both the HAP and the ABF while preserving the liver graft. After 18 months of treatment, the hepatic artery remains patent.

Although the rarity of both complications hinders prospective randomized studies, it is essential to reach a consensus on treating these rare and dangerous complications in liver transplantation. We believe that prospective observational and controlled studies comparing surgical repair results with the available endovascular techniques should be the next step in this regard.

## Conclusions

The appearance of a pseudoaneurysm of the hepatic artery in a liver implant is a rare but potentially fatal complication. The occurrence of an ABF and hemobilia secondary to a HAP is anecdotal. Treatment of both complications using a covered coronary stent allowed a minimally invasive endovascular repair and proper control of the upper GI bleeding, also allowing long-term liver implant preservation.

## Data Availability

Data sharing does not apply to this article as no datasets were generated or analyzed during the current study.

## Abbreviations

HAP: Hepatic artery pseudoaneurysm
ABF: Arterio-biliary fistula
ERCP: Endoscopic retrograde cholangiopancreatography
CT: Computed tomography
